# Ecological transcriptomics of lake-type and riverine sockeye salmon (*Oncorhynchus nerka*)

**DOI:** 10.1186/1472-6785-11-31

**Published:** 2011-12-02

**Authors:** Scott A Pavey, Ben JG Sutherland, Jong Leong, Adrienne Robb, Kris von Schalburg, Troy R Hamon, Ben F Koop, Jennifer L Nielsen

**Affiliations:** 1 National Park Service, Katmai National Park; PO Box 7; King Salmon, AK 99613, USA; 2 Department of Biological Sciences, Simon Fraser University; 8888 University Dr.; Burnaby, BC V5B 1S6 Canada; 3 Centre for Biomedical Research, University of Victoria, 3800 Finnerty Rd.; Victoria, BC V8W 3N5, Canada; 4 USGS Retired, 5817 Yeazell Rd KpS, Longbranch, WA 98351, USA

## Abstract

**Background:**

There are a growing number of genomes sequenced with tentative functions assigned to a large proportion of the individual genes. Model organisms in laboratory settings form the basis for the assignment of gene function, and the ecological context of gene function is lacking. This work addresses this shortcoming by investigating expressed genes of sockeye salmon (*Oncorhynchus nerka*) muscle tissue. We compared morphology and gene expression in natural juvenile sockeye populations related to river and lake habitats. Based on previously documented divergent morphology, feeding strategy, and predation in association with these distinct environments, we expect that burst swimming is favored in riverine population and continuous swimming is favored in lake-type population. In turn we predict that morphology and expressed genes promote burst swimming in riverine sockeye and continuous swimming in lake-type sockeye.

**Results:**

We found the riverine sockeye population had deep, robust bodies and lake-type had shallow, streamlined bodies. Gene expression patterns were measured using a 16K microarray, discovering 141 genes with significant differential expression. Overall, the identity and function of these genes was consistent with our hypothesis. In addition, Gene Ontology (GO) enrichment analyses with a larger set of differentially expressed genes found the "biosynthesis" category enriched for the riverine population and the "metabolism" category enriched for the lake-type population.

**Conclusions:**

This study provides a framework for understanding sockeye life history from a transcriptomic perspective and a starting point for more extensive, targeted studies determining the ecological context of genes.

## Background

The field of genomics is expanding rapidly with full genome and transcriptome sequencing of many model and non-model species. Annotating these genomes continues to pose a challenge [1]. Due to sequence conservation of functional genes and the rapidly growing molecular knowledge of model organisms, basic local alignment search tools (e.g. BLAST) facilitate the initial annotation of non-model genomes [2]. However, the ecological context of genes largely remains a mystery; nearly all gene annotation is based on studies of few model organisms in laboratory environments [3-6]. Thus, genes that function primarily in natural settings remain unannotated, and other genes with known function in laboratory organisms have no ecological context.

Studies of fishes are leading the way in providing an ecological context to genomes [e.g., lake whitefish [*Coregonus clupeaformis *[7,8]], Atlantic salmon [*Salmo salar *[9-11]], killifish [*Fundulus heteroclitus *[12-14]], threespine stickleback [*Gasterosteus aculeatus *[15,16]] and sockeye salmon [*Oncorhynchus nerka *[17-19]]]. These studies have employed three basic methods to relate gene transcription to ecological systems [20]. First is *in situ *gene expression analysis [e.g. [7,19]]. Sampling occurs in the ecological context of interest in nature; fish capture and RNA preservation occur in the field. This method measures both genetic and environmental effects on the transcriptome and it is often not possible to assign gene expression differences to either source. A study design with replication reporting parallel expression differences between two systems reduces population and perhaps environment specific gene expression [e.g. [7]]. In general, this method is applicable to many species including large or long-lived species where laboratory rearing or genetic crosses are not practical. Second, one can remove the natural environmental effect and only test for genetic effects on gene expression in common garden experiments. This strategy compares transcriptomes of genetically distinct ecotypes in controlled conditions [e.g. [21]] and is generally applied to species that can be reared artificially. Reaction norms may be tested by experimentally manipulating conditions. Third, one can perform expression quantitative trait loci (eQTL) analyses by crossing genetically distinct ecotypes in a laboratory setting and mapping gene expression phenotypes to linkage groups [e.g. [22,23]]. This method requires artificial rearing and is only practical for species with short generation times; however, this is also the only method of these three able to determine the overall genomic architecture of gene expression [20]. Unfortunately, the latter two methods are removed from natural environment variation, and therefore may miss heritable expression that requires certain environmental conditions to manifest.

Juvenile sockeye salmon exhibit a life-history dichotomy in their freshwater rearing environments; lake-type populations rear in lakes for one to two years before travelling to the ocean to feed whereas riverine populations rear in river habitats for up to two years [in the riverine subset "sea-type", individuals go to sea before the first winter, whereas "river-type" spend at least one winter in the riverine habitat; [24,25]]. Foraging, water current, and predation differ between habitats [26,27]. Body shape differs between these life history types in association with the environment. In southwest Alaska, riverine sockeye exhibit a deep robust body whereas lake-type sockeye are more fusiforme [27]. This may be the result of both predation regime and a foraging strategy favoring burst swimming in riverine and continuous swimming in lake-type habitats. Similar morphological and behavioral differences are apparent within and among different species of Pacific salmon [28-31].

A set of recent studies characterized the transcriptome in ecotypes of another salmonid, the lake whitefish, employing both the *in situ *and common garden approaches in dwarf and normal ecotypes [7,21]. The primary ecological trade-off between an increase in growth and fecundity in the normal ecotype is the increased energetics in the dwarf ecotype [7,32,33]. The dwarf ecotype exhibits continuous swimming for feeding and is subjected to high predation compared to the normal ecotype [7,33]. Therefore, both continuous swimming during plankton foraging and burst swimming during predator avoidance are likely favored in the dwarf ecotype, resulting in an energy expenditure for metabolism at the expense of growth [32,34]. This trade-off results in great differences in growth rate, age at maturity, body shape, and maximum lifespan.

Phenotypically, lake whitefish ecotypes have drastically different sizes at the same age [34]. Although the size distributions of the sockeye populations in this study overlap at the juvenile life stage, the riverine sockeye are longer and have a more robust body shape [27]. With less extreme morphological differences in ecotypes of sockeye salmon juveniles, we expect the molecular trade-offs to be different from the lake whitefish studies. We expect genes differentially expressed to reflect the differing emphasis on continuous swimming for lake-type and burst swimming for riverine.

In this study, we compare the body morphology and *in situ *transcriptome of two sockeye salmon populations in the same drainage that exhibit these divergent life histories. Differences in foraging strategy and predation may have led to genetic differences between these populations [35]. We expect the transcriptome to reflect the functional molecular trade-offs driven by the ecological differences in these life histories. A greater understanding of the molecular mechanisms that relate to functional ecology will enhance our understanding of the phenotypic diversity of this species, as well as place specific gene annotations into an ecological context.

## Results

### Morphology

**Figure 1:**
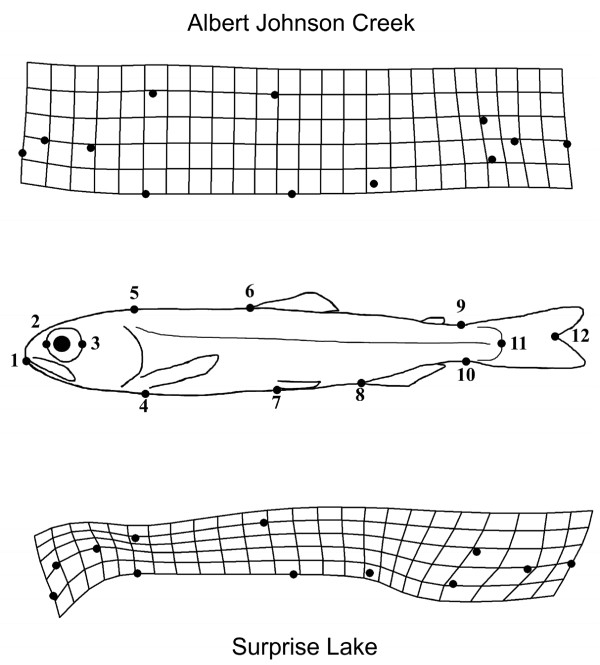
**Landmarks used and deformation grids of Albert Johnson Creek (AJC) and Surprise Lake (SL) fish**. The middle panel depicts the locations of the twelve landmarks used in the geometric morphometric comparison of body shape. the top and bottom panels depect the details of the shape differences of these populations from ecolgically different habitat between AJC (top) and SL (bottom).

Albert Johnson Creek (AJC) juvenile sockeye were differently shaped compared to the Surprise Lake (SL) population, indicated in the significant population term of our model (df = 19/348; F = 6.10; *P *< 0.001). Generally, SL juveniles were streamlined compared with the robust shape of AJC juveniles (Figure 1). The interaction term centroid size × population was not significant and therefore not included in the final model.

### Microarray expression profiles

The microarray analysis indicates 141 transcripts with significant differential expression (average fold change (FC) ≥ 1.5) between individuals from the riverine and lake populations (t-test with Benjamini Hochberg FDR multiple test correction [MTC]; *P *≤ 0.05). Of these, 81 were over-expressed in AJC compared with SL (Table 1) while 60 were over-expressed in SL compared with AJC (Table 2). The fold differences were modest, most of which were below two-fold. In AJC, the genes with the highest over-expression with corrected *P* values were: type II keratin E1 (Genbank:CB510619; FC 2.1; *P *= 0.033), kinesin-like protein (Genbank:CB491150; FC 2.1 *P *= 0.033), and CCAAT/enhancer-binding protein (Genbank:CA050914; FC 1.9; *P *= 0.044). In SL the genes with the highest over-expression were: structural maintenance of chromosomes protein 1B (Genbank:CB488712; FC 2.9; *P *= 0.024), CD81 antigen (Genbank:CA039936; FC 2.76; *P *= 0.033), collagen alpha-2(I) chain precursor (Genbank:CB515159; FC 2.36, *P *= 0.028), ferritin, heavy subunit (Genbank:CB505886; FC 2.33, *P *= 0.044), and troponin I, slow skeletal muscle (Genbank:CB509964; FC 2.29; *P *= 0.039). Overall, the cGRASP annotation file matched our checking of EST sequences in Megablast and tBLASTx quite well, but 6 of 99 genes from the differentially expressed lists with distinct descriptions (not containing words like "unknown" or "predicted") were found to have different descriptions (Additional File 1, Table S1), many of which have been submitted to NCBI databases recently.

**Table 1:** Genes significantly over-expressed in Albert Johnson Creek (AJC) sockeye salmon muscle compared to Surprise Lake (SL)

Function	Fold Change (Up in AJC)	*P *Value Raw	*P *Value MTC	Normalized AJC	StdDev AJC	Normalized SL	StdDev SL	Genbank	Description
Cell division	1.518	0.003	0.033	1.160	0.447	0.765	0.261	CA059189	Golgi reassembly-stacking protein 2
Cell division	1.678	0.008	0.043	1.504	0.936	0.896	0.389	CA060603	Katanin p80 WD40-containing subunit B1
Cell division	1.778	0.011	0.044	1.592	0.849	0.895	0.593	CB515443	Nuclear autoantigenic sperm protein
Cell structure	2.118	0.003	0.033	1.422	1.252	0.671	0.361	CB510619	Oncorhynchus mykiss mRNA for type II keratin E1 (E1 gene)
Development	1.644	0.002	0.033	1.162	0.439	0.707	0.297	CA052389	Plexin-A3 precursor
Development	1.755	0.004	0.036	1.122	0.516	0.639	0.354	CB498195	Reticulon-4
DNA Replication	1.738	0.007	0.042	1.107	0.631	0.637	0.335	CA053921	Girdin
DNA Replication	1.722	0.003	0.033	1.535	0.863	0.892	0.375	CB494447	Poly [ADP-ribose] polymerase 14
Energetic Metabolism	1.601	0.014	0.050	1.287	0.662	0.804	0.413	CA042459	Salmo salar Na, K-ATPase alpha subunit isoform 1b/ii (ATP1A1B/ii)
Energetic Metabolism	1.796	0.013	0.048	1.262	0.885	0.703	0.436	CA064415	Methionine-R-sulfoxide reductase
Energetic Metabolism	1.685	0.007	0.042	1.552	0.764	0.921	0.482	CB503498	Creatine kinase, testis isozyme
Immune	1.597	0.009	0.044	1.182	0.732	0.740	0.256	CA048654	Oncorhynchus mykiss SYPG1 MHCII-alpha and Raftlin-like pseudogenes
Immune	1.893	0.002	0.033	1.293	0.663	0.683	0.392	CA063034	Transient receptor potential cation channel subfamily M member 4
Immune	1.722	0.003	0.033	1.096	0.548	0.636	0.293	CB492871	Oncorhynchus mykiss mRNA for MHC class II alpha (onmy-DAA*02 gene)
Iron Binding	1.540	0.012	0.046	1.134	0.400	0.736	0.372	CB494485	Ferritin, heavy subunit
Iron Binding	1.657	0.004	0.033	0.981	0.449	0.592	0.263	CB501208	Hemoglobin subunit alpha-4
Mitochondria	1.873	0.007	0.043	1.428	0.953	0.763	0.472	CB489874	TIM21-like protein, mitochondrial precursor
Mitochondria	1.528	0.001	0.033	1.092	0.365	0.714	0.217	CB498084	NADH dehydrogenase [ubiquinone] 1 alpha subcomplex subunit 4
Protein folding	1.644	0.006	0.041	1.210	0.548	0.736	0.359	CA052325	Prefoldin subunit 2
Protein transport	1.911	0.003	0.044	1.095	0.697	0.573	0.284	CA051435	Ras-related protein Rab-14
Protein transport	1.674	0.006	0.041	1.237	0.702	0.739	0.323	CA054426	ADP-ribosylation factor 1
Protein transport	2.103	0.001	0.033	1.263	0.669	0.600	0.366	CB491150	Kinesin-like protein KIF20A
Signal	1.544	0.003	0.033	1.174	0.510	0.760	0.235	CB514723	G-protein coupled receptor APJ homolog
Transcription factor (neg)	1.652	0.011	0.044	1.301	0.489	0.787	0.479	CB499801	Pre-B-cell leukemia transcription factor 2
Transcription regulation	1.974	0.009	0.044	1.335	1.062	0.676	0.460	CA050914	CCAAT/enhancer-binding protein delta
Transcription regulation	1.903	0.002	0.033	1.210	0.667	0.636	0.348	CB498272	Nucleolar protein 5A
Translation	1.518	0.001	0.033	1.067	0.301	0.703	0.217	CA037622	60S ribosomal protein L36
Translation	1.967	0.003	0.033	1.196	0.714	0.608	0.381	CA045500	60S ribosomal protein L9
Translation	1.528	0.001	0.033	1.297	0.504	0.849	0.226	CA055741	60S ribosomal protein L9
Translation	1.613	0.001	0.033	1.252	0.544	0.776	0.250	CA770261	60S ribosomal protein L23
Translation	1.538	0.013	0.048	0.944	0.433	0.614	0.285	CA770402	60S ribosomal protein L15
Translation	1.538	0.007	0.042	1.069	0.256	0.695	0.336	CB493600	40S ribosomal protein S30
Translation	1.711	0.002	0.033	1.377	0.739	0.805	0.306	CB493907	40S ribosomal protein S19
Translation	1.632	0.006	0.040	1.137	0.434	0.697	0.352	CB503205	60S ribosomal protein L4-B
Translation	1.719	0.009	0.044	0.908	0.529	0.528	0.289	CB514402	60S ribosomal protein L19
Translation	1.717	0.014	0.050	1.555	1.267	0.905	0.376	CK990280	60S acidic ribosomal protein P1
Transmembrane	1.536	0.006	0.042	1.285	0.591	0.836	0.310	CA041663	pfam07690, MFS_1, Major Facilitator Superfamily
Many functions	1.526	0.002	0.033	1.206	0.366	0.791	0.296	CA047582	Somatotropin precursor
Many functions	1.502	0.011	0.044	1.125	0.407	0.749	0.338	CA052412	Ectodysplasin-A
Many functions	1.630	0.010	0.044	1.154	0.613	0.708	0.350	CA061568	Nucleophosmin
Many functions	1.695	0.014	0.050	1.326	0.799	0.782	0.451	CB503191	Anterior gradient protein 2 homolog precurser
Many functions	1.614	0.011	0.044	1.147	0.668	0.711	0.309	CB515428	Tartrate-resistant acid phosphatase type 5 precursor
Other functions	1.762	0.003	0.033	1.234	0.769	0.701	0.287	CA044554	Prothymosin alpha
Other functions	1.677	0.004	0.033	1.283	0.734	0.765	0.290	CA055729	Transmembrane protein 178 precursor
Other functions	1.507	0.010	0.044	1.002	0.406	0.665	0.286	CA058231	Antolefinin
Other functions	1.518	0.006	0.041	1.141	0.647	0.752	0.128	CA059713	Probable RNA-directed DNA polymerase from transposon BS
Other functions	1.842	0.011	0.044	1.594	0.965	0.865	0.599	CB490454	Zona pellucida sperm-binding protein 3 precursor
Other functions	1.630	0.003	0.033	1.401	0.569	0.860	0.369	CB492596	Proteasome subunit alpha type 4
Other functions	1.614	0.008	0.044	1.164	0.582	0.721	0.333	CB494074	Oncorhynchus mykiss clone Glan 1 transposon e
Other functions	1.574	0.012	0.046	1.333	0.796	0.847	0.323	CB497649	Nucleoside diphosphate kinase B
Other functions	1.528	0.012	0.046	1.212	0.637	0.793	0.301	CB498610	UNKNOWN
Other functions	1.640	0.003	0.033	1.289	0.622	0.786	0.305	CB499656	Dual specificity mitogen-activated protein kinase kinase 6
Other functions	1.775	0.005	0.040	1.266	0.692	0.713	0.389	CB511161	Voltage-dependent anion-selective channel protein 2
Other functions	1.752	0.007	0.043	1.073	0.533	0.613	0.365	CB511880	Serine/threonine-protein kinase Haspin
Other functions	1.513	0.001	0.033	1.061	0.316	0.701	0.221	CB514112	Serine/threonine/tyrosine-interacting protein
Other functions	1.536	0.005	0.038	1.238	0.600	0.806	0.250	CB514435	Williams-Beuren syndrome chromosome region 16 protein homolog
Unknown	1.657	0.011	0.044	1.156	0.416	0.698	0.430	CA039908	UNKNOWN
Unknown	1.538	0.002	0.033	1.402	0.577	0.912	0.258	CA039963	UNKNOWN
Unknown	1.765	0.001	0.033	1.627	0.789	0.921	0.367	CA040487	PREDICTED: similar to Keratin-associated protein 10-1
Unknown	1.674	0.010	0.044	1.113	0.748	0.665	0.284	CA041505	UNKNOWN
Unknown	1.607	0.003	0.033	1.212	0.436	0.754	0.338	CA050842	UNKNOWN
Unknown	1.715	0.005	0.040	1.368	0.802	0.798	0.361	CA051475	UNKNOWN
Unknown	1.723	0.003	0.033	1.307	0.686	0.758	0.326	CA054597	UNKNOWN
Unknown	1.565	0.003	0.033	1.001	0.430	0.639	0.218	CA057262	PREDICTED: similar to Rsbn1 protein [Danio rerio]
Unknown	1.872	0.000	0.033	1.204	0.261	0.643	0.321	CA058259	UNKNOWN
Unknown	1.537	0.011	0.044	1.350	0.696	0.878	0.346	CA058522	UNKNOWN
Unknown	1.698	0.004	0.033	0.956	0.321	0.563	0.304	CA061924	UNKNOWN
Unknown	1.891	0.012	0.047	1.581	1.414	0.836	0.484	CB490094	UNKNOWN
Unknown	1.558	0.005	0.040	1.143	0.493	0.734	0.294	CB492336	UNKNOWN
Unknown	1.634	0.001	0.033	1.291	0.479	0.790	0.280	CB492905	UNKNOWN
Unknown	1.660	0.008	0.044	1.254	0.653	0.755	0.380	CB494690	UNKNOWN
Unknown	1.695	0.001	0.033	1.297	0.534	0.765	0.281	CB497128	UNKNOWN
Unknown	1.816	0.009	0.044	1.431	0.997	0.788	0.448	CB500083	UNKNOWN
Unknown	1.730	0.010	0.044	1.218	0.459	0.704	0.476	CB509719	PREDICTED: similar to CC chemokine SCYA103 [Danio rerio]
Unknown	1.693	0.010	0.044	1.243	0.634	0.734	0.417	CB510709	UNKNOWN
Unknown	1.626	0.002	0.033	1.366	0.590	0.840	0.317	CB511393	UNKNOWN
Unknown	1.603	0.008	0.043	1.193	0.606	0.744	0.327	CB511789	PREDICTED: similar to Protein C14orf159, mitochondrial precursor
Unknown	1.619	0.013	0.047	1.195	0.818	0.738	0.271	CB513822	UNKNOWN
Unknown	1.604	0.011	0.044	1.273	0.687	0.794	0.367	CB514071	UNKNOWN
Unknown	1.605	0.009	0.044	1.069	0.625	0.666	0.263	CK990538	UNKNOWN
Unknown	1.501	0.008	0.043	1.158	0.590	0.772	0.227	CK991114	UNKNOWN

Genes with significantly greater expression in Albert Johnson Creek (AJC) sockeye salmon muscle compared to Surprise Lake (SL) sockeye salmon muscle. We present the gene description, the raw *P *values, the multiple test corrected *P *values, the average normalized expression values and standard deviations for each population, the Genbank ID and gene description.

**Table 2:** Genes significantly over-expressed in Surprise Lake (SL) sockeye salmon muscle compared to Albert Johnson Creek.

Function	Fold change (Up in SL)	*P *value raw	*P *Value MTC	Normalized AJC	StdDev AJC	Normalized SL	StdDev SL	Genbank	Description
Aerobic infrastructure	2.208	0.002	0.024	0.667	0.465	1.474	1.039	CB510651	72 kDa type IV collagenase precursor
Cell interactions	2.755	0.004	0.033	0.625	0.571	1.720	2.017	CA039936	CD81 antigen
Cell interactions	1.698	0.003	0.028	0.648	0.303	1.100	0.497	CB488646	Cysteine-rich protein 1
Cell interactions	1.543	0.002	0.023	0.777	0.307	1.199	0.377	CB498736	Cysteine-rich protein 1
Cell interactions	2.358	0.003	0.028	0.764	0.687	1.803	1.383	CB515159	Collagen alpha-2(I) chain precursor
DNA	1.610	0.001	0.020	0.789	0.314	1.272	0.425	CA051642	Ribonucleoside-diphosphate reductase M2 subunit
DNA	2.907	0.002	0.024	0.658	0.399	1.910	2.344	CB488712	Structural maintenance of chromosomes protein 1B
DNA	2.252	0.000	0.005	0.706	0.288	1.589	0.805	CB490371	Histone H3.3
Energetic Metabolism	1.825	0.006	0.036	0.836	0.275	1.527	1.051	CA041073	Calcium/calmodulin-dependent protein kinase type II delta chain
Energetic Metabolism	1.508	0.005	0.036	0.792	0.363	1.194	0.376	CA049006	SUMO-activating enzyme subunit 2
Energetic Metabolism	1.534	0.001	0.020	0.703	0.293	1.078	0.280	CA061459	cAMP-dependent protein kinase, beta-2-catalytic subunit
Energetic Metabolism	1.553	0.001	0.020	0.828	0.308	1.286	0.396	CA064428	6-phosphogluconate dehydrogenase, decarboxylating
Energetic Metabolism	1.684	0.003	0.028	0.902	0.325	1.518	0.759	CB498472	Selenide, water dikinase 2
Iron Binding	2.326	0.010	0.044	0.730	0.661	1.697	1.751	CB505886	Ferritin, heavy subunit
Lipid catabolism	1.580	0.004	0.033	0.806	0.271	1.273	0.578	CA038195	Phospholipase A2, acidic 1 precursor
Mitochondria	2.053	0.003	0.028	0.861	0.707	1.766	0.930	CA056742	Carnitine O-palmitoyltransferase 2, mitochondrial precursor
Mitochondria	2.083	0.008	0.039	0.680	0.336	1.416	1.308	CA058136	5-aminolevulinate synthase, nonspecific, mitochondrial precursor
Mitochondria	1.745	0.010	0.044	0.683	0.382	1.192	0.710	CA060285	Adenylate kinase isoenzyme 2, mitochondrial
Mitochondria	1.623	0.005	0.036	0.877	0.354	1.424	0.682	CA062017	Single-stranded DNA-binding protein, mitochondrial precursor
Muscle contraction regulation	2.294	0.007	0.039	0.674	0.486	1.548	1.579	CB509964	Troponin I, slow skeletal muscle
Muscle contraction regulation	1.613	0.010	0.044	0.807	0.386	1.300	0.650	CB510901	Troponin I, slow skeletal muscle
Organelle movement	1.672	0.001	0.020	0.735	0.228	1.230	0.557	CB498105	Tubulin alpha-1C chain
Protein breakdown	1.504	0.005	0.035	0.827	0.409	1.245	0.307	CK990590	Trypsin precursor
Protein breakdown	1.520	0.006	0.037	0.815	0.369	1.238	0.436	CK991067	Cathepsin H precursor
Protein transport	1.575	0.012	0.047	0.836	0.365	1.316	0.662	CA042407	Protein disulfide-isomerase A6 precursor
Protien transport	1.570	0.001	0.020	0.683	0.322	1.072	0.211	CA044589	Protein transport protein Sec61 subunit beta
Sugar binding	1.515	0.008	0.039	0.799	0.309	1.211	0.504	CB505852	serum lectin isoform 3 precursor [Salmo salar]
Transcription (neg)	1.961	0.012	0.047	0.690	0.480	1.352	1.070	CA054647	Selenoprotein K
Transcription regulation	1.592	0.008	0.039	0.739	0.336	1.176	0.540	CB494071	14-3-3-like protein GF14-F
Translation	1.590	0.001	0.020	0.685	0.340	1.088	0.223	CA061402	Pseudouridylate synthase 7 homolog
Translation	1.721	0.000	0.005	0.753	0.242	1.294	0.407	CB516915	Eukaryotic translation initiation factor 3 subunit 7
Transport	2.037	0.003	0.027	0.687	0.444	1.400	0.874	CB496796	Transmembrane emp24 domain-containing protein 3 precursor
Transport	1.880	0.009	0.044	0.704	0.449	1.323	0.920	CB512385	Vacuolar protein sorting-associated protein 41 homolog
Many functions	1.704	0.000	0.002	0.725	0.209	1.235	0.311	CA048664	Protein C-ets-1
Many functions	1.845	0.000	0.010	0.685	0.241	1.264	0.520	CA050496	Transitional endoplasmic reticulum ATPase
Many functions	1.678	0.006	0.037	0.882	0.447	1.479	0.729	CB515873	Dihydropyrimidinase
Other functions	1.855	0.006	0.037	0.858	0.439	1.593	1.058	CA044775	UNKNOWN
Other functions	2.105	0.006	0.037	0.739	0.420	1.556	1.360	CA051433	Transmembrane and ubiquitin-like domain-containing protein 2
Other functions	2.016	0.005	0.035	0.812	0.480	1.638	1.205	CA056436	UNKNOWN
Other functions	1.645	0.000	0.018	0.880	0.292	1.449	0.514	CA056626	Lithognathus mormyrus clone lmos2p08h02 mRNA sequence
Other functions	1.502	0.001	0.020	0.790	0.284	1.187	0.321	CA062511	Translocon-associated protein subunit alpha precursor
Other functions	1.976	0.004	0.033	0.579	0.396	1.144	0.695	CB498745	KH domain-containing, RNA-binding, signal transduction-associated protein 1
Other functions	1.570	0.012	0.049	0.878	0.241	1.379	0.785	CB516580	Kelch-like protein 6
Other functions	1.692	0.004	0.033	0.851	0.335	1.440	0.741	CB516919	Extracellular matrix protein 1 precursor
Unknown	1.536	0.010	0.044	0.838	0.420	1.289	0.512	CA040470	UNKNOWN
Unknown	1.789	0.001	0.020	0.723	0.286	1.294	0.650	CA041684	UNKNOWN
Unknown	1.623	0.007	0.039	0.881	0.511	1.429	0.555	CA054094	UNKNOWN
Unknown	1.656	0.001	0.020	0.747	0.326	1.237	0.420	CA058336	PREDICTED: similar to Transforming growth factor, beta-induced [Danio rerio] >
Unknown	1.563	0.001	0.020	0.629	0.224	0.982	0.332	CA060640	UNKNOWN
Unknown	1.634	0.008	0.039	0.879	0.454	1.437	0.670	CA767983	PREDICTED: similar to small inducible cytokine SCYA105 [Danio rerio]
Unknown	1.534	0.001	0.020	0.863	0.332	1.324	0.362	CB492594	UNKNOWN
Unknown	1.541	0.008	0.039	0.782	0.296	1.204	0.544	CB493987	UNKNOWN
Unknown	1.808	0.004	0.033	0.709	0.484	1.282	0.571	CB502569	UNKNOWN
Unknown	1.575	0.009	0.044	0.855	0.372	1.346	0.640	CB505933	UNKNOWN
Unknown	1.585	0.002	0.024	0.793	0.395	1.257	0.322	CB506647	UNKNOWN
Unknown	1.517	0.010	0.044	1.005	0.575	1.527	0.437	CB515453	UNKNOWN
Unknown	1.543	0.010	0.044	0.758	0.338	1.168	0.517	CB516051	UNKNOWN
Unknown	1.727	0.001	0.020	0.837	0.479	1.445	0.424	CB516202	Sterile alpha motif domain-containing protein 9-like
Unknown	1.570	0.007	0.039	0.828	0.346	1.299	0.578	CK991103	PREDICTED: similar to transposase [Strongylocentrotus purpuratus]
Unknown	1.751	0.000	0.010	0.712	0.280	1.246	0.433	CK991281	UNKNOWN

Genes with significantly greater expression in Surprise Lake sockeye salmon muscle compared to Albert Johnson Creek (AJC) sockeye salmon muscle. We present the gene description, the raw *P *values, the multiple test corrected *P *values, the average normalized expression values and standard deviations for each population, the Genbank ID and gene description.

In order to expand differential expression lists to facilitate functional analysis, the stringent multiple test correction was removed during significance testing, and transcripts that showed any expression difference were included in this analysis (*P *≤ 0.05). As a result, 1026 genes were found significantly differentially expressed in muscle. Of these, 498 genes were expressed at higher (or over-expressed) levels in AJC compared with SL. Of these over-expressed transcripts, 230 and 240 were annotated with biological process and molecular function Gene Ontology (GO) terms, respectively. In all cases we used the GO Slim dataset. In biological process, biosynthesis (GO:9058; *P *= 0.009) and behavior (GO:7610; P = 0.019) were the only GO categories significantly enriched (Table 3). In the molecular function ontology, the only enriched category was structural molecular activity (GO:5198; 43 genes, *P *< 0.001).

**Table 3:** Gene Ontology (GO) enrichment results

Category	*P *value	Genes in GO category over-expressed	% of differentially expressed genes in GO category	Genes in GO category on array	% genes on array in GO category
**Over-expressed in AJC: Biological process**					
GO:9058: biosynthesis	0.009	52	24.41	1264	17.82
GO:7610: behavior	0.019	10	4.695	156	2.2
**Over-expressed in AJC: Molecular function**					
GO:5198: structural molecule activity	< 0.001	43	17.92	750	9.043
**Over-expressed in SL: Biological process**					
GO:8152: metabolism	0.019	192	71.91	4674	65.91
**Over-expressed in SL: Molecular function**					
GO:16209: antioxidant activity	0.013	6	1.917	52	0.627
GO:8135: translation factor activity, nucleic acid binding	0.010	13	4.153	166	2.001
GO:45182: translation regulator activity	0.014	13	4.153	173	2.086
GO:5489: electron transporter activity	0.005	17	5.431	225	2.713
GO:8233: peptidase activity	0.043	28	8.946	529	6.378
GO:3824: catalytic activity	0.001	166	53.04	3645	43.95

These are the significant GO Slim categories representing both biological process and molecular function ontologies for population specific significantly over-expressed (*P *≤ 0.05; no multiple test correction) features. For each significant GO category, we include the *P *value number of over-expressed genes in that GO, percentage of representation in the over-expressed list, number of features of that GO in the microarray, and percentage of representation on the entire microarray.

We found 528 genes significantly expressed at higher levels in SL muscle compared to AJC (*P *< 0.05, no MTC). Of this list, 267 and 313 features were annotated with biological process and molecular function GO terms, respectively. In this analysis, metabolism (GO:8152) was the only biological process category significantly enriched (*P *= 0.019), containing 192 genes. There are six significant categories enriched from the molecular function category (Table 3).

### Reverse-Transcription Quantitative Polymerase Chain Reaction (RT-qPCR)

**Figure 2:**
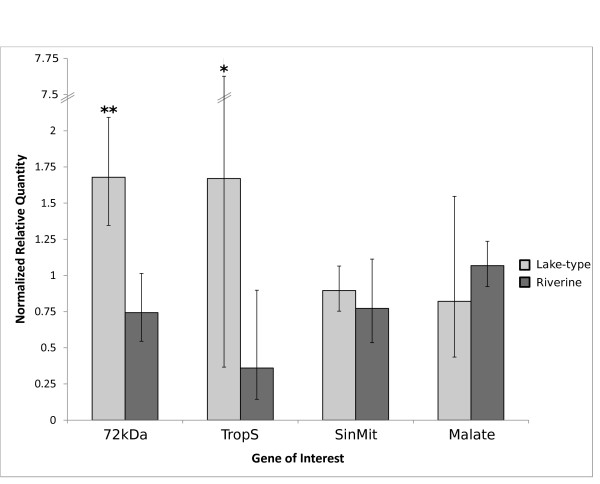
**RT-qPCR transcript profiles displaying mean normalized quantities in the lake-type and riverine ecotypes**. "72 kDa" is 72 kDa type IV collagenase precursor (Genbank:CB510651), "TropS" is troponin I, slow skeletal muscle (Genbank:CB510901), and "SinMit" is single-stranded DNA-binding protein, mitochondrial precursor (Genbank:CA062007). Expression is relative to the geometric mean of expression levels of normalizers 40S ribosomal and 5-aminolevulinate synthase (Genbank:CB493907 and CA058136). Malate dehydrogenase (Genbank:CA044864) is included as an example a feature that was not significantly differentially regulated with the microarray analysis. Significance was determined by a one-tailed Mann-Whitney *U *test, *denotes p ≤ 0.05 **denotes p ≤ 0.001.

As 40s ribosomal and 5-amino-levuleninic acid synthase (Genbank:CB493907 and CA058136) were identified as the most stable normalizer candidates through the geNORM algorithm, these transcripts were used to generate relative expression ratios of genes of interest (GOI). The four GOI's are 72 kDa type IV collagenase precursor (Genbank:CB510651), troponin I, slow skeletal muscle (Genbank:CB510901), single-stranded DNA-binding protein, mitochondrial precursor (Genbank:CA062007), and malate dehydrogenase (Genbank:CA044864). Two of the four investigated genes were differentially expressed, 72 kDa type IV collagenase precursor and troponin I, slow skeletal muscle, both significantly over-expressed in SL juveniles, presented in Figure 2. 72 kDa type IV collagenase precursor was highly significant (FC > 2; p = 0.00013). Troponin I, slow skeletal muscle displayed a high level of biological variation among biological replicates, as can be viewed by the large 95% confidence intervals for this GOI (Figure 2).

## Discussion

We have characterized molecular phenotypes in muscle tissue that relate to morphology, life history, and ecology in sockeye salmon. We also discovered differentially expressed genes and enriched functional categories associated with differing morphology and life history types of sockeye salmon in two habitats. This work represents the first characterization of a molecular phenotype in muscle or any other tissue of juvenile sockeye between these common habitat types. Because these populations are in relatively pristine habitats, these ecologically based gene expression differences provide a reference for published and future studies of sockeye salmon in habitats more impacted by human activities [17,36,37]

Riverine sockeye have a deep, robust body compared with the lake-type life history [27]. We find this pattern between the AJC and SL populations (Figure 1). In parallel, some patterns in expression profiles in the present study reflect these phenotypes. For example, in AJC, ten ribosomal proteins were over-expressed compared with SL and one of these (Genbank:CA045500) was among the highest over-expressed in AJC (Table 1). In comparison, we did not identify any ribosomal proteins over-expressed in SL compared with AJC (Table 2). All of these features on the array map to different contigs with the cGRASP Expressed Sequence Tag (EST) clustering online tool and therefore are likely to represent different genes. Many ribosomal proteins stabilize the structure composed mostly of ribosomal RNA [38]. Thus, the differential expression of these ribosomal proteins may indicate more protein synthesis in the muscle tissue of AJC sockeye. In addition, five genes associated with cell division, DNA replication and a growth hormone gene were over-expressed in AJC (Table 1 and Additional File 1, Table S1). These patterns are consistent with faster growth and more muscle mass associated with the deeper body morphology in AJC sockeye [27]. The GO category of biosynthesis (GO:9058) is defined as "The chemical reactions and pathways resulting in the formation of substances; typically the energy-requiring part of metabolism in which simpler substances are transformed into more complex ones" (http://amigo.geneontology.org). This was an enriched category in AJC of the GO Slim biological process ontology which further underscores that the expression profile in AJC corresponds to increased biomolecule production.

Creatine kinase (Genbank:CB503498) was over-expressed in AJC compared with SL. This gene is potentially important in both aerobic respiration in the pathway of oxidative phosphorylation, as well as anaerobic metabolism in glycolosis [39]. However in both processes this gene regulates the amount of available cellular ATP so it facilitates fluctuating energy demands [39]. This may be important to the riverine "wait and burst" feeding style of AJC, which may involve more variable levels of feeding activity.

In the SL gene enrichment analysis, the sole significant generic GO Slim ontology is metabolism (GO:8152). Also, one of the significant GO terms in the molecular function ontology is electron transport indicating aerobic respiration. Many of the individual genes in the over-expressed list relate to energy metabolism, mitochondria, and muscle contraction regulation. This is compatible with increased metabolism, especially for continuous swimming. Several of these genes may be particularly important for the continuous swimming strategy of lake-type sockeye. Troponin I, slow skeletal muscle (present twice in the over-expressed list; Genbank:CB509964 and CB510901) is a gene that regulates muscle contraction and the "slow" label of the annotation indicates that this transcript is specific to slow twitch or aerobic muscle fiber [40]. We confirmed with the cGRASP EST clustering database that these features map to different contigs and therefore likely represent two different genes. The latter of these two genes was also found to be significantly over-expressed in our RT-qPCR analysis (Figure 2). These findings could be the result of either increased red muscle fibers present, increased recruitment of red muscle fiber, or both. Additionally, 72 kDa type IV collagenase precursor (Genbank:CB510651), was over-expressed in SL juveniles and is implicated in blood vascular remodelling [41]. This gene was also found to be over-expressed in our qPCR analysis. These may lay the infrastructure for increased aerobic needs. Another SL over-expressed gene, selenoprotein K (Genbank:CA054647), is a response to oxidative stress [42], which may occur in increased aerobic activity. We did not separate red and white muscle tissue in our experiment. Many fish species have the muscle fiber types distinctly separated and ecotypes may differ in their composition of red and white muscle sections [43]. Pacific salmon, however, have red muscle fibers mixed in with the main white muscle mass [44]. We collected all of the main locomotion muscle tissue from each individual as we also wanted to capture gene expression differences due to different muscle fiber composition.

Other significant GO terms have less of a clear functional relationship with the ecology of these populations. The translation factor activity (GO:8135 and GO:45182) terms represented in SL is composed of translation initiation factor genes. This is in contrast to over-expression of ribosomal proteins in AJC including 10 in the significantly over-expressed genes (Table 1) and 37 of the 52 genes in the biosynthesis (GO:9058) term (Table 3). It is also unclear why behavior (GO:7610) is a significant GO term enriched in AJC (Table 3). There are likely important behavioral differences between these populations, but the ten genes contained within this GO term, appear to be genes that have many divergent functions and the behavioural annotations are mostly related to mice.

RT-qPCR results were concordant to the microarray results in three out of four cases. Additionally, in all four cases, the average expression level was in the same direction for the RT-qPCR and microarray assays (Additional File 2, Table S2). Our sample size was smaller with the RT-qPCR (SL: n = 9 and AJC n = 10) and this may have resulted in a reduction of power compared with the microarray assay and the lack of significance agreement in one of the four comparisons.

An unanticipated discovery was the increased expression of immune function genes in AJC, including two features annotated with MHC II function (Genbank:CB492871 and CA048654; Table 1). This may indicate differing immunity challenges in the river and lake rearing habitats of this study. This finding is a good example of indirect hypothesis generation that can come from using such large data-set producing tools. As microarrays facilitate the screening of a large number of genes they may uncover unexpected traits that are difficult to measure, even if not identified as potential traits of interest during experimental design [20].

We detected differential regulation of select regulatory genes over-expressed in AJC including two transcription factors. Pro-B-cell leukemia transcription factor 2 (Genbank:CB499801) and CCAAT/enhancer-binding protein delta (Genbank:CA050914) regulate transcription [45]. The latter has the second highest over-expression fold change in AJC. In SL, one gene annotated as "unknown" in the cGRASP annotation file, was identified through the re-BLAST methods as "far upstream binding element protein 3" (Entrez Gene ID 100194998). These regulatory genes could have cascading effects in gene expression [46], and their roles in these ecotypes should be investigated further.

Whether due to recent sequence submissions [47], or through the challenges of assembling large EST datasets in individuals with recent genome duplications [48,49], a few annotations of differentially expressed genes varied from the originally released 16K annotation file [50]. The genes with new annotation can be viewed in Additional File 1, Table S1. One example that our individual BLAST efforts identified was 60 S ribosomal protein L14 (BT060370.1). Also, we identified another gene as the antifreeze protein, type 2 ice-structuring protein (Entrez Gene ID: 100195780). This gene has obvious ecological implications for the colonization of new lakes and may have been especially important in post-glacial lakes. These two different BLAST hits were very recently annotated 25-August 2010 [47]. These few difference between the cGRASP annotation file and current blast hits underscore both the computational complexity of assembling genomes and the constantly changing knowledge of gene function.

Both lists of differentially expressed transcripts contain many unknown function annotations, and although we cannot assign any molecular function to these genes based on this study, we do now have ecological context for these genes. Furthermore, as more genes are annotated, we may gain more insight on the role of these unknowns in the ecotype variances, as was the case with "far upstream binding element protein 3" as described above.

Our results yield both similarities and differences when compared to the gene expression work on lake whitefish [7,21]. The morphological and expected ecological differences in juvenile sockeye salmon are not nearly as extreme as those observed in lake whitefish, which are drastically different in growth rate and age of maturity. However, like the present study, the fold change differences between ecotypes in the lake whitefish work with both microarrays and qPCR are modest, suggesting this may be the norm for ecological transcriptomic differences in natural populations. Unlike lake whitefish, sockeye salmon are anadromous, and our study populations move to the ocean after freshwater rearing, where feeding environments and access to them may be similar [51]. Therefore, differences at the juvenile rearing stage may be limited, because this is only one part of a complex life history, and the life history types may developmentally converge for the ocean feeding stage.

In lake whitefish, parvalbumin beta (Genbank:AF538283) was the only gene involved with muscle contraction regulation that was consistently over-expressed in the dwarf ecotype. We did not find evidence of over-expression of this gene in SL, but another gene involved with muscle contraction, the slow twitch isomer of troponin, was significantly over-expressed in SL. It is expected that feeding strategy promotes continuous swimming in dwarf lake whitefish [34]. In addition, dwarf lake whitefish are under high predation compared with normal whitefish, an ecological attribute responsible for increased burst swimming. This should favor both aerobic and anaerobic metabolism in the same ecotype resulting in selection favoring overall increased metabolism and muscle contraction [34]. In the present study, high predation and a burst swimming feeding strategy are expected only in riverine AJC, whereas a continuous swimming strategy and low predation should occur in lake-type SL. These differing scenarios of selection may result in less-pronounced partitioning of swimming energetics in sockeye salmon compared with lake whitefish.

In lake whitefish, many of the differentially expressed genes in nature retained differential expression when individuals were raised in a common environment [21]. Also, gene mis-expression in lake whitefish dwarf × normal backcross is associated with reduced egg survival [52]. It is difficult to distinguish cause from effect in these situations, as the mis-expression in underdeveloped eggs may be the result of the underdeveloped phenotype and the cause may be in an unmeasured earlier stage of development [53]. In summary, in lake whitefish, gene expression traits have a genetic component and can affect traits important to reproduction.

Other fish species also manifest the benthic/limnetic ecotypes including threespine stickleback [54], Dolly Varden (*Salvelinus malma*) [55] and Arctic charr (*S. alpinus*) [56]. Though despite many behavioral, morphological, and genetic studies, relatively few of these important ecological model species have been investigated at the transcriptomic level. Elmer et al. [57] found non-synonymous divergence in ESTs related to biosynthesis, metabolism and development in South American crater lake cichlids (*Amphilophus astorquii *and *A. zaliosus*). Other studies of fish transcriptomics have focused on spawning survivorship [36] and salt/freshwater transitions [37,58]

Our study has limitations in that we only present a single tissue type in a single point in time for these populations. Also, the morphological sampling and the gene expression sampling took place in different years, though we expect that the morphological differences are temporally stable, at least in the time scale between the two sampling periods. The morphological and gene expression differences between these populations may be due to phenotypic plasticity, adaptive or non-adaptive genetic processes, or a combination of all three [12,59]. Like many phenotypic traits, gene expression is an integration of both environmental and genetic components [20,60]. Phenotypic plasticity itself may have a genetic component and may be adaptive, especially in species with range expansion and contraction, where colonization of new habitats occurs often [61,62]. Even gene expression differences that are purely plastic are important to further our understanding of ecology and colonization, and may facilitate adaptation in other non-plastic traits [63].

## Conclusions

We have developed the first dataset characterizing gene expression differences between two populations of sockeye salmon representing lake-type and riverine life histories. Although this represents a first step in considering the ecological transcriptomic differences of juvenile sockeye, we have already identified clear patterns relating to the divergent ecological phenotypes of these populations. In riverine sockeye muscle tissue, genes of higher expression were primarily associated with growth whereas in the lake-type sockeye, metabolism was the theme. Since these populations reside in a pristine part of the sockeye range, this study may serve as a reference location for future studies of populations that are more impacted by human activities.

## Methods

### Study site

**Figure 3:**
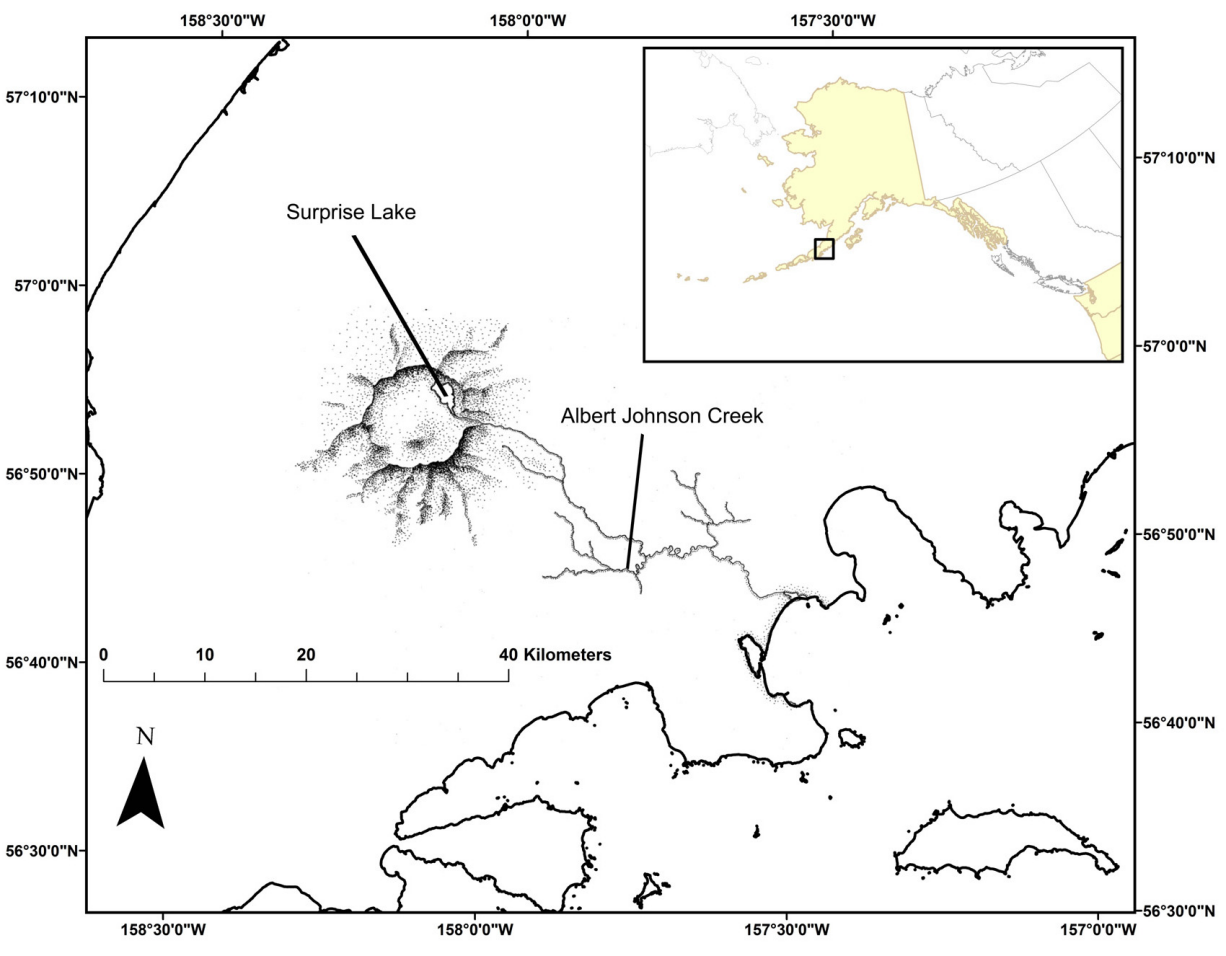
**The two sampling locations of this study showing Albert Johnson Creek (AJC) and Surprise Lake (SL) in Aniakchak Caldera**.

Aniakchak National Monument and Preserve (ANMP) in southwest Alaska provides a unique system to study these sockeye life history strategies (Figure 3). The ANMP has undergone several recent geologic events. A massive volcanic eruption 3,650 years before present (b.p.) formed a large caldera (Aniakchak Caldera) that filled with water creating a lake [64,65]. Approximately 1,800 b.p. [66] the caldera wall collapsed resulting in a large flood and the formation of the Aniakchak River, which connects the remainder of the caldera lake (Surprise Lake; elevation 321 m) with the Pacific Ocean through "The Gates", a chasm opened through the caldera wall by the flood [67]. A large fluvial plain was established when the passing flood dropped sediment as it exited the caldera. Several smaller eruptions have occurred, including well-documented events approximately 500 and 80 b.p. [64]. Sometime after the 500 b.p. eruption lake-type sockeye salmon colonized Surprise Lake (SL) and used the lake for juvenile rearing [68]. A riverine sockeye population also rears in Albert Johnson Creek (AJC), the largest tributary of Aniakchak River [35]. Albert Johnson Creek is a low gradient stream that meets Aniakchak River at the base of the volcano in the large fluvial plain that was the result of the caldera draining flood, 1,800 b.p.. Thus, current populations representing each of lake-type (SL) and riverine (AJC) life history types coexist in the same drainage.

### Morphology methods

In order to make a morphological comparison between the two populations in this study, we reanalyzed a subset of the morphological dataset from Pavey et al. 2010 of 360 age 0 (meaning previous to first winter) juvenile sockeye with only the two current study populations [Table 1 in [27]]. Twelve landmarks were digitized on each image using TpsDig (Figure 1, middle panel). All methods were identical except here we only compared the SL and AJC populations. All uses of animals in this study were approved by the animal care and use committee of either Simon Fraser University or University of Alaska Anchorage.

### Gene expression methods

Juvenile sockeye salmon were sampled on August 8^th ^2007. The time of sampling for Albert Johnson Creek was 1535 h to 1703 h and Surprise Lake was 1832 h to 2057 h. The entire sampling effort took place within 5.5 hours including transportation from Albert Johnson Creek to Surprise Lake by a Cessna 185 airplane. A beach seine was used to capture fish and a strict sampling protocol including sampling time was enforced to reduce fish-to-fish sampling bias. Fish of similar lengths were sampled from each site. Mean fork length was 45.9 mm (n = 17; SD = 3.5 mm) for AJC and 45.0 mm (n = 13; SD = 5.6 mm) for SL. One fish from each seine haul was placed in a lethal solution of MS-222 (100 mg/l). An incision was made in the body cavity with a scalpel and the entire fish was placed in RNA*later*™ (Ambion). The maximum time between netting a fish to RNA preservation was five minutes. The samples were kept cool in the field and transported, then frozen, and stored at -20°C to -80°C until RNA extraction.

#### RNA preparation

The samples were thawed and blotted with a Kimwipe^®^. All of the primary locomotion muscle tissue including red and white muscle tissue was removed from each fish. Total RNA was extracted with a modified protocol of the Invitrogen TRIzol^® ^Plus RNA purification kit using PureLink™ Micro-to-Midi™ columns. Disruption and homogenization were achieved with a MixerMill MM301 (Retsch). The manufacturer's protocol was followed for each extraction with the exception of using 150 μl of chloroform and 150 μl of low pH phenol to ensure dissociation of proteins and isolation of RNA. The quality of all RNA samples was verified on a 1% agarose gel. All samples were quantified with a Spectrophotometer ND-1000 (NanoDrop).

#### cDNA and aRNA synthesis and labeling

cDNA was synthesized with Invitrogen SuperScript™ III Indirect cDNA labeling system kits per manufacturer's instructions. In brief, 10 μg of total RNA from a single individual was combined with a master mix including reverse-transcriptase and oligo (dT)_20 _primers. This reaction was incubated for three hours at 46°C to synthesize single-stranded cDNA. The samples were cleaned with the S.N.A.P ™ column purification procedure (Invitrogen).

A reference pool was prepared with representative total RNA samples of juvenile sockeye muscle and liver, and adult sockeye brain, muscle and liver. Multiple tissues were used to ensure hybridization of the reference channel to all spots that may have been hybridized by the sample channel cDNA, and therefore able to be quantified as a ratio. The total RNA was amplified using an Amino Allyl MessageAmp™ II aRNA amplification Kit (Ambion AM1753) as per manufacturers instructions. Briefly, RNA from several individuals from both populations of a single tissue type was combined. Then, single-stranded cDNA was synthesized from the RNA whereupon the second strand was synthesized with DNA polymerase. This product was purified through columns, and then amino allyl-modified aRNA was transcribed from the cDNA. The aRNA from divergent tissue types was combined in equal amounts at this point and this common reference pool was labeled with Cy3 to be compared with a single individual in the experiment labeled with Cy5.

Individual samples and reference material was coupled with mono-reactive CyDye™ packs (GE Healthcare). In short, the common reference pool of aRNA was coupled with Cy3 and the individual sockeye muscle tissue cDNA with Cy5 dyes for one hour at 4°C. The samples were then purified to remove all uncoupled dye using S.N.A.P.™ columns as per manufacturer's instructions (Invitrogen). The dye coupled sample and reference were stored at 4°C in the dark until hybridization.

#### Microarray hybridization

We used the cGRASP 16K cDNA microarray to compare the transcriptomes of these populations with divergent life histories. This microarray consists of 16,006 elements chosen from 300,000 Atlantic salmon and rainbow trout cDNA libraries [50]. The libraries were derived from a variety of tissue types at different development stages, and conditions. Element sequences were chosen for minimum overlap, sequence quality, and other criteria [50].

We followed an established hybridization protocol for the 16 k cDNA array [50]. In brief, both 250 ng of reference aRNA and 500 ng of sample cDNA were collected in a single tube and kept dark. The mixture was concentrated with a speed vacuum and brought up to 23 μl with RNase free water (Gibco). Hybridization buffer #3 (Ambion) was heated to 65°C while occasionally mixing for one hour. The heated buffer and LNAdt blocker (Genesphere) was then added to the collected sample, as per manufacturer's instructions. We used the Tecan HS 4800 Pro, an automated hybridization machine to hybridize sample cDNA to the arrays (Tecan). Before the sample injection, the programmed Tecan washed with several solutions containing first 1 × SSC, then 0.1 × SSC 0.014% SDS, then 5 × SSC, 0.01% SDS, and 0.2% BSA. Samples were heated to 80°C for 5 minutes, and then kept at 65°C until injected onto the pre-washed arrays in a Tecan HS 4800 Pro, as per manufacturers' instructions. Microarrays were hybridized for 16 hours, and the full protocol for the hybridization can be viewed in Additional File 3, Table S3.

Post-hybridization, arrays were rinsed in the Tecan modules with increasingly stringent SSC and SDS solutions, starting with 2 × SSC, 0.014%SDS for four washes incrementally decreasing temperature, then one final wash of 0.2 × SSC at 23 °C. Finally, slides were dried with 37 psi nitrogen gas and kept dark until scanned. Current protocols for cGRASP microarrays are available at: http://web.uvic.ca/grasp/microarray/protocols/tecan_hybridization_protocol.pdf

#### Scanning and quantifying

All microarrays were scanned immediately after hybridization was complete using a ScanArray Express (Perkin-Elmer). The microarray images were quantified manually with ImaGene 5.6.1 (BioDiscovery). Spots with unusual morphology, offset, or other poor quality parameters were flagged as marginal and excluded these from downstream analyses.

#### Array normalization and statistical analysis

We performed all analyses in GeneSpring GX 7.3 (Agilent). The arrays were normalized as per typical two-color experiments by performing an array-wide intensity-dependent Lowess normalization, followed by a per gene normalization, which normalized each spot to the median value. The average base/proportionate value was calculated to be an intensity of 72, so we filtered data to retain only the 14,652 entities with average raw signal expression values greater than 72 in at least one of the populations. This became our base expression data for analysis. Our GeneSpring analysis was performed in two ways. First, the dataset was filtered to retain only the genes where the average differential expression was ≥ 1.5 fold. This list was used in a t-test without equal variances assumption (*P *≤ 0.05; no equal variance assumption) with a Benjamini and Hochberg False Discovery Rate multiple test correction (MTC; [69]. The spot ID's from the cGRASP 16 k annotation file (current annotation files available at: http://web.uvic.ca/grasp/microarray/array.html; [48]) were used to associate ESTs on the array with gene descriptions. To confirm current annotation of the differential gene list, a Megablast was performed on associated EST sequences, or used tBLASTx on the associated EST, or contiguous sequence (contig). The default parameters were used for these database queries. All normalized expression values as well as raw data was deposited in the NCBI Gene Expression Omnibus database (GEO Accession: GSE31214).

#### Gene Ontology analysis

To account for all genes potentially differentially expressed, not just those with high fold changes, or that passed highly stringent statistical methods such as those that passed the multiple test correction, a less stringent filtering on the base gene list was generated for the Gene Ontology (GO) analysis. Genes significantly differentially expressed by any amount that passed a t-test (*P *≤ 0.05) and without a multiple test correction were included. We then performed GO enrichment analysis on this list of over-expressed genes using the GO browser in GeneSpring. GO categories that were significantly represented at a higher proportion in the over-expressed list than the array at large (*P *≤ 0.05) for GO Generic Slim ontology of both biological process and molecular function were produced.

#### Reverse-transcription Quantitative Polymerase Chain Reaction (RT-qPCR)

Total RNA samples used in microarray analysis were used for RT-qPCR. Single-stranded cDNA was synthesized from 4 μg total RNA using SuperScript^® ^III First-Strand Synthesis System for RT-PCR (Invitrogen), as per manufacturer's instructions. Briefly, total RNA was incubated for 50 min at 50°C with 5 μM oligo(dT_20_) primers. Each sample was then diluted 10 fold to prepare for qPCR. Four genes of interest (GOI) were selected for potential ecological relevance. Amplicons were designed within 500 base pairs of the 3' end of the coding sequence for each GOI in conserved regions between Atlantic salmon and rainbow trout (*O. mykiss*), and checked for specificity of sequence by BLAST.

Primer efficiency was tested by a standard curve of experimental sample cDNA synthesized as described above. The standard curve was generated from an initial 10-fold diluted sample which was then used as the starting point for a two-fold, 6 point serial dilution series. qPCR amplification was performed with SYBR GreenER™ qPCR SuperMix Universal master mix, as per manufacturer's instructions (Invitrogen), in 20 μL reaction volumes containing 400 μM primers on an Mx3000P™ thermal cycler (Agilent) with the following thermal regime: 95°C for 7 min (1 cycle); 95°C for 30 s, 60°C for 1 min, 72°C for 30 s (40 cycles); followed by a melt curve of 95°C for 30 s reading at every 0.5°C increment. Singularity and correct product identification was determined by agarose gel electrophoresis, melt curve analysis, and amplicon sequencing. Primer sequences, correlation with dilution series (R^2^), and efficiency values are presented in Table 4.

**Table 4:** Gene descriptions, primer sequences, efficiency, and R^2^ for each gene quantified with RT-qPCR.

Description	Primers 5'-3' (forward, reverse)	PCR efficiency, %	**R**^**2**^
72 kDa type IV collagenase	5' TTC GCT GGA GAC AAG TTC TG 5' TTT GAC GAT CTT CAG GCT ACT G	80.1	0.99
Troponin 1 Slow	5' CAG GAC TTA GGA GGG AAG TTT AAG 5' AGA CAT GGC CTC CAC ATT CTT AC	84.0	0.99
Single Mit Prec	5' AGA TGT CAG CCA GAA GAC GAC 5' TCG AAT GTT GTC GCT TAA GAA TAC	105.3	0.99
40S Ribosome	5' CGA GAA GTG GTT CTA CAC CAG AG 5' GGT TCT TCT CCA CCA TCT TGA G	95.1	0.99
Malate dehydogenase	5' ATT TCT ACA GTG CAG AGA GG 5' GAA CAG GGA ATG AGT AGA TGA GG	106.5	0.99
5-amino-levuleninic acid synthase	5' ACA TCA TCC CTG TCA GAG TGT C 5' TGA TTG GGA CTT GAG AGG TAG AC	95.6	0.97

For each GOI, biological replicates were run in quadruplicate on one plate with 9 and 10 biological replicates for lake-type and riverine ecotype conditions, respectively. Clear outlier technical replicates (> 0.2 Ct values from the other replicates) were removed from analysis. If one biological replicate had two technical replicates indicating one Ct value and the other two indicated a different Ct value, none were removed, as the correct pair could not be discerned. The replicate variability was within 0.5 Ct for 110 of 114 sample-target combinations. All NTC did not indicate the melt temperature of the GOI amplicon, and 5 of 6 investigated genes had more than 7 Ct between the average NTC Ct and the most dilute unknown sample (troponin I, slow skeletal muscle was only 3.8 Ct from the average NTC primer dimer; SABiosciences). Additionally, all GOIs were contained within the standard curve dilution series, with the exception of one malate dehydogenase sample which was more dilute than the most dilute point of the dilution series, and troponin I, slow skeletal muscle, which contained 3 samples more concentrated than the dilution series (all within 1.5 Ct of the most concentrated), and 8 samples less concentrated than the dilution series (all within 2 Ct of the least concentrated).

Data analysis was performed using qbasePLUS (Biogazelle). All quantified genes were tested as normalizer candidates using geNORM. We did not include Single mitochondial precurser in this test as it appeared to be co-regulated with 5-aminolevulinate synthase in these samples (both transcripts are mitochondrial precursors and showed similar non-normalized expression patterns (*data not shown*)). The most stably-expressed transcripts were 40S ribosomal and 5-amino-levuleninic acid synthase, collectively displaying an M value of 0.645 and coefficient of variation of 0.225, within limits typically observed for stably expressed reference genes in heterogeneous samples (M value ≤ 1 and CV ≤ 0.5) [70]. Additionally, malate dehydogenase was identified as the third best candidate, and although it was not used for normalization, it was identified as non-significantly differentially expressed element in the microarray results.

Normalized relative quantities were tested for normality through an Anderson-Darling test (Minitab 16). Not all GOIs were found to display normally distributed expression ratios, and therefore a non-parametric, one-tailed Mann-Whitney *U *test was used to determine significance of fold change between the groups (a one-tailed test was selected as the directionality was expected from microarray results).

## Authors' contributions

SAP wrote the paper, performed the fieldwork, designed the study, performed the gene expression lab work, and data analysis. BJGS co-wrote the paper and performed qPCR data analysis. JL performed the bioinformatics and gene annotation. AR optimized cDNA synthesis and microarray hybridization protocols. KVS optimized RNA extraction protocols. TH co-designed the study. BFK provided facilities, personnel and contributed to study design. JLN conceived the study. All authors have read and approve of the final manuscript.

## Supplementary Material

Additional file 1 **Update of six gene annotations that were differentially expressed**. Updated gene annotations for the six gene descriptions that differed substantially from the original annotation file and our use of megaBLAST and tBLASTx. Click here for file

Additional file 2 **Comparison of fold change results between microarray and RT-qPCR assays**. Fold change values comparison between microarray and RT-qPCR assays. Bold values indicate statistically significantly different from 1. The full descriptions of the genes are: 72 kDa type IV collagenase precursor (Genbank:CB510651), troponin I, slow skeletal muscle (Genbank:CB510901), single-stranded DNA-binding protein, mitochondrial precursor (Genbank:CA062007), and malate dehydrogenase (Genbank: CA044864). Click here for file

Additional file 3 **Tecan HS 400 Pro hybridization protocol**. Detailed description of all microarray hybridization steps. Click here for file

## References

[B1] Reeves GA Talavera D Thornton JM Genome and proteome annotation: organization, interpretation and integration J R Soc Interface 2009 6 129 147 10.1098/rsif.2008.0341 19019817 PMC2658791

[B2] Rokas A Abbot P Harnessing genomics for evolutionary insights Trends Ecol Evol 2009 24 192 200 10.1016/j.tree.2008.11.004 19201503

[B3] Pena-Castillo L Hughes TR Why are there still over 1000 uncharacterized yeast genes? Genetics 2007 176 7 14 10.1534/genetics.107.074468 17435240 PMC1893027

[B4] Landry CR Aubin-Horth N Ecological annotation of genes and genomes through ecological genomics Mol Ecol 2007 16 4419 4421 10.1111/j.1365-294X.2007.03504.x 17883391

[B5] McKay JK Stinchcombe JR Ecological genomics of model eukaryotes Evolution 2008 62 2953 2957 10.1111/j.1558-5646.2008.00536.x 19055681

[B6] Kammenga JE Herman MA Ouborg NJ Johnson L Breitling R Microarray challenges in ecology Trends Ecol Evol 2007 22 273 279 10.1016/j.tree.2007.01.013 17296243

[B7] Derome N Duchesne P Bernatchez L Parallelism in gene transcription among sympatric lake whitefish (*Coregonus clupeaformis *Mitchill) ecotypes Mol Ecol 2006 15 1239 1249 10.1111/j.1365-294X.2005.02968.x 16626451

[B8] Derome N Bernatchez L The transcriptomics of ecological convergence between 2 limnetic coregonine fishes (Salmonidae) Mol Biol Evol 2006 23 2370 2378 10.1093/molbev/msl110 16963516

[B9] Aubin-Horth N Landry CR Letcher BH Hofmann HA Alternative life histories shape brain gene expression profiles in males of the same population Proc R Soc B 2005 272 1655 1662 10.1098/rspb.2005.3125 16087419 PMC1559854

[B10] Aubin-Horth N Letcher BH Hofmann HA Interaction of rearing environment and reproductive tactic on gene expression profiles in Atlantic salmon J Hered 2005 96 261 278 10.1093/jhered/esi030 15653555

[B11] Roberge C Guderley H Bernatchez L Genomewide identification of genes under directional selection: Gene transcription Q_ST _scan in diverging Atlantic salmon subpopulations Genetics 2007 177 1011 1022 10.1534/genetics.107.073759 17720934 PMC2034609

[B12] Whitehead A Crawford DL Neutral and adaptive variation in gene expression Proc Natl Acad Sci USA 2006 103 5425 5430 10.1073/pnas.0507648103 16567645 PMC1414633

[B13] Oleksiak MF Churchill GA Crawford DL Variation in gene expression within and among natural populations Nat Genet 2002 32 261 266 10.1038/ng983 12219088

[B14] Scott CP Williams DA Crawford DL The effect of genetic and environmental variation on metabolic gene expression Mol Ecol 2009 18 2832 2843 10.1111/j.1365-294X.2009.04235.x 19500250 PMC2705469

[B15] Chan YF Villarreal G Marks M Shapiro M Jones F Petrov D Dickson M Southwick A Absher D Grimwood J From trait to base pairs: Parallel evolution of pelvic reduction in three-spined sticklebacks occurs by repeated deletion of a tissue-specific pelvic enhancer at *Pitx1* Mech Dev 2009 126 S14 S15

[B16] Coyle SM Huntingford FA Peichel CL Parallel evolution of *Pitx1 *underlies pelvic reduction in Scottish threespine stickleback (*Gasterosteus aculeatus*) J Hered 2007 98 581 586 10.1093/jhered/esm066 17693397

[B17] Eliason EJ Clark TD Hague MJ Hanson LM Gallagher ZS Jeffries KM Gale MK Patterson DA Hinch SG Farrell AP Differences in thermal tolerance among sockeye salmon populations Science 2011 332 109 112 10.1126/science.1199158 21454790

[B18] Martins EG Hinch SG Patterson DA Hague MJ Cooke SJ Miller KM Lapointe MF English KK Farrell AP Effects of river temperature and climate warming on stock-specific survival of adult migrating Fraser River sockeye salmon (Oncorhynchus nerka) Global Change Biol 2011 17 99 114 10.1111/j.1365-2486.2010.02241.x

[B19] Miller KM Li SR Kaukinen KH Ginther N Hammill E Curtis JMR Patterson DA Sierocinski T Donnison L Pavlidis P Genomic signatures predict migration and spawning failure in wild Canadian salmon Science 2011 331 214 217 10.1126/science.1196901 21233388

[B20] Pavey SA Collin H Nosil P Rogers SM The role of gene expression in ecological speciation Ann N Y Acad Sci 2010 1206 110 129 10.1111/j.1749-6632.2010.05765.x 20860685 PMC3066407

[B21] St-Cyr J Derome N Bernatchez L The transcriptomics of life-history trade-offs in whitefish species pairs (*Coregonus *sp.) Mol Ecol 2008 17 1850 1870 10.1111/j.1365-294X.2008.03696.x 18312278

[B22] Whiteley AR Derome N Rogers SM St-Cyr J Laroche J Labbe A Nolte A Renaut S Jeukens J Bernatchez L The phenomics and expression quantitative trait locus mapping of brain transcriptomes regulating adaptive divergence in lake whitefish species pairs (*Coregonus *sp.) Genetics 2008 180 147 164 10.1534/genetics.108.089938 18757926 PMC2535670

[B23] Derome N Bougas B Rogers SM Whiteley AR Labbe A Laroche J Bernatchez L Pervasive sex-linked effects on transcription regulation as revealed by expression quantitative trait loci mapping in lake whitefish species pairs (*Coregonus *sp., Salmonidae) Genetics 2008 179 1903 1917 10.1534/genetics.107.086306 18660540 PMC2516068

[B24] Wood CC Life history variation and population structure in sockeye salmon Am Fish Soc Symp 1995 17 195 216

[B25] Beacham TD McIntosh B Macconnachie C Population structure of lake-type and river-type sockeye salmon in transboundary rivers of northern British Columbia J Fish Biol 2004 65 389 402 10.1111/j.0022-1112.2004.00457.x

[B26] Murphy ML Heifetz J Thedinga JF Johnson SW Koski KV Habitat utilization by juvenile Pacific salmon (*Oncorhynchus*) in the glacial Taku River, southeast Alaska Can J Fish Aquat Sci 1989 46 1677 1685 10.1139/f89-213

[B27] Pavey SA Nielsen JL Mackas RH Hamon TR Breden F Contrasting ecology shapes juvenile lake-type and riverine sockeye salmon Trans Am Fish Soc 2010 139 1584 1594 10.1577/T09-182.1

[B28] Swain DP Holtby LB Differences in morphology and behavior between juvenile coho salmon (*Oncorhynchus kisutch*) rearing in a lake and in its tributary stream Can J Fish Aquat Sci 1989 46 1406 1414 10.1139/f89-180

[B29] Scott WB Crossman EJ Freshwater fishes of Canada J Fish Res Board Can 1973 184

[B30] Taylor EB McPhail JD Variation in body morphology among British Columbia populations of coho salmon, *Oncorhynchus kisutch* Can J Fish Aquat Sci 1985 42 2020 2028 10.1139/f85-249

[B31] Hoar WS The behaviour of juvenile pacific salmon, with particular reference to the sockeye (*Oncorhynchus nerka*) J Fish Res Board Can 1954 11 69 97 10.1139/f54-009

[B32] Trudel M Tremblay A Schetagne R Rasmussen JB Why are dwarf fish so small? An energetic analysis of polymorphism in lake whitefish (*Coregonus clupeaformis*) Can J Fish Aquat Sci 2001 58 394 405 10.1139/f00-252

[B33] Rogers SM Gagnon C Bernatchez L Genetically based phenotype-environment association for swimming behavior in lake whitefish ecotypes Evolution 2002 56 2322 2329 12487361 10.1111/j.0014-3820.2002.tb00155.x

[B34] Lu G Bernatchez L Correlated trophic specialization and genetic divergence in sympatric lake whitefish ecotypes (*Coregonus clupeaformis*): support for the ecological speciation hypothesis Evolution 1999 53 1491 1505 10.2307/2640895 28565561

[B35] Pavey SA Hamon TR Nielsen JL Revisiting evolutionary dead ends in sockeye salmon (*Oncorhynchus nerka*) life history Can J Fish Aquat Sci 2007 64 1199 1208 10.1139/f07-091

[B36] Miller KM Li S Kaukinen KH Ginther N Hammill E Curtis JMR Patterson DA Sierocinski T Donnison L Pavlidis P Genomic signatures predict migration and spawning failure in wild canadian salmon Science 2011 331 214 217 10.1126/science.1196901 21233388

[B37] Miller KM Schulze AD Ginther N Li SR Patterson DA Farrell AP Hinch SG Salmon spawning migration: Metabolic shifts and environmental triggers Comp Biochem Physiol D Genomics Proteomics 2009 4 75 89 10.1016/j.cbd.2008.11.002 20403740

[B38] Alberts B Alexander J Lewis J Raff M Roberts K Walter P Molecular Biology of the Cell 2002 New York: Taylor & Francis Group

[B39] Wallimann T Wyss M Brdiczka D Nicolay K Eppenberger HM Intracellular compartmentation, structure and function of creatine-kinase isoenzymes in tissues with high and fuctuating energy demands--the phosocreatine circuit for cellular-energy homeostasis Biochem J 1992 281 21 40 1731757 10.1042/bj2810021 PMC1130636

[B40] Corin SJ Juhasz O Zhu L Conley P Kedes L Wade R Structure and expression of the human slow twitch skeletal muscle troponin I gene J Biol Chem 1994 269 10651 10659 8144655

[B41] Fernandez-Patron C Radomski MW Davidge ST Vascular matrix metalloproteinase-2 cleaves big endothelin-1 yielding a novel vasoconstrictor Circul Res 1999 85 906 911 10.1161/01.res.85.10.906 10559137

[B42] Lu C Qiu F Zhou H Peng Y Hao W Xu J Yuan J Wang S Qiang B Xu C Peng X Identification and characterization of selenoprotein K: An antioxidant in cardiomyocytes FEBS Lett 2006 580 5189 5197 10.1016/j.febslet.2006.08.065 16962588

[B43] Johnston IA Abercromby M Vieira VLA Sigursteindottir RJ Kristjansson BK Sibthorpe D Skulason S Rapid evolution of muscle fibre number in post-glacial populations of Arctic charr *Salvelinus alpinus* J Exp Biol 2004 207 4343 4360 10.1242/jeb.01292 15557021

[B44] Altringham J Ellerby D Fish swimming: patterns in muscle function J Exp Biol 1999 202 3397 3403 10562522 10.1242/jeb.202.23.3397

[B45] Wang H Peiris TH Mowery A Le Lay J Gao Y Greenbaum LE CCAAT/Enhancer binding protein-{beta} is a transcriptional tegulator of peroxisome-proliferator-activated receptor-{gamma} coactivator-1{alpha} in the regenerating liver Mol Endocrinol 2008 22 1596 1605 10.1210/me.2007-0388 18467525 PMC2453599

[B46] Davis-Smyth T Duncan RC Zheng T Michelotti G Levens D The far upstream element-binding proteins comprise an ancient family of single-strand DNA-binding transactivators J Biol Chem 1996 271 31679 31687 10.1074/jbc.271.49.31679 8940189

[B47] Leong JS Jantzen SG von Schalburg KR Cooper GA Messmer AM Liao NY Munro S Moore R Holt RA Jones SJM *Salmo salar *and *Esox lucius *full-length cDNA sequences reveal changes in evolutionary pressures on a post-tetraploidization genome BMC Genomics 2010 11 10.1186/1471-2164-11-279 PMC2886063 20433749

[B48] Koop BF von Schalburg KR Leong J Walker N Lieph R Cooper GA Robb A Beetz-Sargent M Holt RA Moore R A salmonid EST genomic study: genes, duplications, phylogeny and microarrays BMC Genomics 2008 9 595 10.1186/1471-2164-9-595 19014685 PMC2628678

[B49] Li LS Khuri S Valafar F, Valafar H A comparison of DNA fragment assembly algorithms METMBS '04: Proceedings of the International Conference on Mathematics and Engineering Techniques in Medicine and Biological Sciences 2004 329 335

[B50] von Schalburg KR Rise ML Cooper GA Brown GD Gibbs AR Nelson CC Davidson WS Koop BF Fish and chips: Various methodologies demonstrate utility of a 16,006-gene salmonid microarray BMC Genomics 2005 6 126 10.1186/1471-2164-6-126 16164747 PMC1239916

[B51] Burgner RL Groot C, Margolis L Life history of sockeye salmon Pacific Salmon Life Histories 1991 Vancouver: University of British Columbia 3 117

[B52] Renaut S Nolte AW Bernatchez L Gene expression divergence and hybrid misexpression between lake whitefish species pairs (*Coregonus *spp. Salmonidae) Mol Biol Evol 2009 26 925 936 10.1093/molbev/msp017 19174479

[B53] Noor MAF Feder JL Speciation genetics: Evolving approaches Nat Rev Genet 2006 7 851 861 17033626 10.1038/nrg1968

[B54] Willacker JJ Von Hippel FA Wilton PR Walton KM Classification of threespine stickleback along the benthic-limnetic axis Biol J Linn Soc 2010 101 595 608 10.1111/j.1095-8312.2010.01531.x PMC3017379 21221422

[B55] Ostberg CO Pavlov SD Hauser L Evolutionary relationships among sympatric life history forms of Dolly Varden inhabiting the landlocked Kronotsky Lake, Kamchatka, and a neighboring anadromous population Trans Am Fish Soc 2009 138 1 14 10.1577/T08-016.1

[B56] Malmquist HJ Snorrason SS Skulason S Jonsson B Sandlund OT Jonasson PM Diet differentiation in polymorphic arctic charr in Thingvallavatn, Iceland J Anim Ecol 1992 61 21 35 10.2307/5505

[B57] Elmer KR Fan S Gunter HM Jones JC Boekhoff S Kuraku S Meyer A Rapid evolution and selection inferred from the transcriptomes of sympatric crater lake cichlid fishes Mol Ecol 2010 19 197 211 20331780 10.1111/j.1365-294X.2009.04488.x

[B58] Giger T Excoffier L Amstutz U Day PJR Champigneulle A Hansen MM Kelso J Largiader CR Population transcriptomics of life-history variation in the genus Salmo Mol Ecol 2008 17 3095 3108 10.1111/j.1365-294X.2008.03820.x 18522696

[B59] Broadley MR White PJ Hammond JP Graham NS Bowen HC Emmerson ZF Fray RG Iannetta PPM McNicol JW May ST Evidence of neutral transcriptome evolution in plants New Phytol 2008 180 587 593 10.1111/j.1469-8137.2008.02640.x 18801004

[B60] Gibson G Weir B The quantitative genetics of transcription Trends Genet 2005 21 616 623 10.1016/j.tig.2005.08.010 16154229

[B61] Ghalambor CK McKay JK Carroll SP Reznick DN Adaptive versus non-adaptive phenotypic plasticity and the potential for contemporary adaptation in new environments Funct Ecol 2007 21 394 407 10.1111/j.1365-2435.2007.01283.x

[B62] Yeh PJ Price TD Adaptive phenotypic plasticity and the successful colonization of a novel environment Am Nat 2004 164 531 542 10.1086/423825 15459883

[B63] Price TD Qvarnstrom A Irwin DE The role of phenotypic plasticity in driving genetic evolution Proc R Soc B 2003 270 1433 1440 10.1098/rspb.2003.2372 12965006 PMC1691402

[B64] McGimsey RG Waythomas CF Neal CA High stand catastrophic draining of intracaldera Surprise Lake, Aniakchak Volcano, Alaska Geologic Studies in Alaska by the U S Geological Survey; U S Geological Survey Bulletin 199412805813

[B65] Pearce NJG Westgate JA Preece SJ Eastwood WJ Perkins WT Identification of Aniakchak (Alaska) tephra in Greenland ice core challenges the 1645 BC date for the Minoan eruption Geochem Geophys Geosyst 2004 5 Q03005

[B66] VanderHoek R Myron R Cultural remains from a catastrophic landscape: an archaeological overview and assessment of Aniakchak National Monument and Preserve Book Cultural remains from a catastrophic landscape: an archaeological overview and assessment of Aniakchak National Monument and Preserve 2004 City: National Park Service (Editor ed.^eds.)

[B67] Hubbard BR A world inside a mountain Natl Geogr Mag 1931 60 319 345

[B68] Pavey SA Hamon TR Nielsen JL Recent ecological divergence despite migration in sockeye salmon (*Oncorhynchus nerka*) Evolution 2010 64 1773 1783 10.1111/j.1558-5646.2009.00927.x 20030707 PMC2901516

[B69] Benjamini Y Yekutieli D The control of the false discovery rate in multiple testing under dependency Ann Stat 2001 29 1165 1188 10.1214/aos/1013699998

[B70] Hellemans J Mortier G De Paepe A Speleman F Vandesompele J qBase relative quantification framework and software for management and automated analysis of real-time quantitative PCR data Genome Biol 2007 8 10.1186/gb-2007-8-2-r19 PMC1852402 17291332

