# Supplementary material for: Ecological transcriptomics of lake-type and riverine sockeye salmon (Oncorhynchus nerka)
Source: BMC Ecol. 2011 Dec 2;11:31. doi: 10.1186/1472-6785-11-31 (PMC3295673; doi:10.1186/1472-6785-11-31)
Supplement: Additional file 1 — Update of six gene annotations that were differentially expressed. Updated gene annotations for the six gene descriptions that differed substantially from the original annotation file and our use of megaBLAST and tBLASTx. [file 1472-6785-11-31-S1.DOC]

### Additional File 1: Table_S1.doc: Update of six gene annotations that were differentially expressed.

Updated gene annotations for the six gene descriptions that differed substantially from the original annotation file and our use of megaBLAST and tBLASTx.

| Direction Over-expressed | cGRASP Genbank | cGRASP Description | BLAST Description |
| --- | --- | --- | --- |
| Up in AJC | CB494485 | Ferritin, heavy subunit | 40s ribosomal protein |
| Up in AJC | CB501208 | Hemoglobin subunit alpha-4 | 60S ribosomal protein L14 |
| Up in AJC | CA047582 | Somatotropin precursor | growth hormone gene |
| Up in SL | CB505886 | Ferritin, heavy subunit | piptidipropyl isomerase |
| Up in SL | CB505852 | serum lectin isoform 3 precursor [Salmo salar] | [Type-2 ice-structuring protein [Salmo salar]](http://www.ncbi.nlm.nih.gov/sites/entrez?db=gene&cmd=search&term=100195780&RID=6B4YVGBE016&log$=geneexplicitnucl&blast_rank=1) |
| Up in SL | CB488712 | Structural maintenance of chromosomes protein 1B | Selenide, water dikinase 1 [Salmo salar] |
