# Supplementary material for: Ecological transcriptomics of lake-type and riverine sockeye salmon (Oncorhynchus nerka)
Source: BMC Ecol. 2011 Dec 2;11:31. doi: 10.1186/1472-6785-11-31 (PMC3295673; doi:10.1186/1472-6785-11-31)
Supplement: Additional file 2 — Comparison of fold change results between microarray and RT-qPCR assays. Fold change values comparison between microarray and RT-qPCR assays. Bold values indicate statistically significantly different from 1. The full descriptions of the genes are: 72 kDa type IV collagenase precursor (Genbank:CB510651), troponin I, slow skeletal muscle (Genbank:CB510901), single-stranded DNA-binding protein, mitochondrial precursor (Genbank:CA062007), and malate dehydrogenase (Genbank: CA044864). [file 1472-6785-11-31-S2.DOC]

### Additional File 2: Table_S2.doc: Comparison of fold change results between microarray and RT-qPCR assays

Fold change values comparison between microarray and RT-qPCR assays. Bold values indicate statistically significantly different from 1. The full descriptions of the genes are: 72 kDa type IV collagenase precursor (Genebank:CB510651), troponin I, slow skeletal muscle (Genbank:CB510901), single-stranded DNA-binding protein, mitochondrial precursor (Genebank:CA062007), and malate dehydogenase (CA044864).

| Gene | Relative fold change | |
| --- | --- | --- |
|  | Microarray | RT-qPCR |
| 72kDa | **2.2** | **2.26** |
| TropS | **2.29** | **4.4** |
| SinMit | **1.62** | 1.06 |
| Malate | 1.49 | 1.29 |
