# Supplementary material for: Ecological transcriptomics of lake-type and riverine sockeye salmon (Oncorhynchus nerka)
Source: BMC Ecol. 2011 Dec 2;11:31. doi: 10.1186/1472-6785-11-31 (PMC3295673; doi:10.1186/1472-6785-11-31)
Supplement: Additional file 3 — Tecan HS 400 Pro hybridization protocol. Detailed description of all microarray hybridization steps. [file 1472-6785-11-31-S3.DOC]

### Additional File 3:Table_S3.doc: Tecan HS 400 Pro hybridization protocol.

Detailed description of all microarray hybridization steps

| Step | Action | Temp.ºC | Solution | Wash/Agitation Time | Soak Time | Times |
| --- | --- | --- | --- | --- | --- | --- |
| 1 | wash | 23 | 0.1xSSC, 0.014%SDS | 0:00:30 | 0:00:30 | 3 |
| 2 | wash | 23 | 0.02xSSC | 0:01:00 | 0:00:30 | 2 |
| 3 | wash | 49 | 5xSSC, 0.01% SDS, 0.2% BSA | 0:01:00 | 0:00:00 | 1 |
| 4 | hybridize | 49 |  | 1:00:00 |  | 1 |
| 5 | wash | 46 | 2xSSC, 0.014%SDS | 0:01:00 | 0:00:30 | 2 |
| 6 | inject sample | 49 |  |  |  |  |
| 7 | hybridize | 49 |  | 1:00:00 |  | 1 |
| 8 | hybridize | 53 |  | 1:00:00 |  | 1 |
| 9 | hybridize | 49 |  | 4:00:00 |  | 1 |
| 10 | hybridize | 53 |  | 1:00:00 |  | 1 |
| 11 | hybridize | 49 |  | 4:00:00 |  | 1 |
| 12 | hybridize | 53 |  | 1:00:00 |  | 1 |
| 13 | hybridize | 49 |  | 4:00:00 |  | 1 |
| 14 | wash | 49 | 2xSSC, 0.014%SDS | 0:01:00 | 0:00:00 | 1 |
| 15 | hybridize | 49 |  | 0:03:00 | 0:00:00 | 1 |
| 16 | wash | 49 | 2xSSC, 0.014%SDS | 0:00:30 | 0:00:00 | 1 |
| 17 | wash | 39 | 2xSSC, 0.014%SDS | 0:00:20 | 0:00:00 | 1 |
| 18 | wash | 30 | 2xSSC, 0.014%SDS | 0:00:20 | 0:00:00 | 1 |
| 19 | wash | 23 | 1xSSC | 0:00:20 | 0:00:00 | 1 |
| 20 | wash | 23 | 0.02xSSC | 0:00:30 | 0:00:00 | 3 |
| 21 | dry | 23 |  | 0:02:30 |  | 1 |
